# Supplementary material for: Structural insights into the formation and voltage degradation of lithium- and manganese-rich layered oxides
Source: Nat Commun. 2019 Nov 26;10:5365. doi: 10.1038/s41467-019-13240-z (PMC6879514; doi:10.1038/s41467-019-13240-z)
Supplement: Supplementary file 1 — Supplementary Information [file 41467_2019_13240_MOESM1_ESM.pdf]

Supplementary Information for

Structural insights into the formation and voltage degradation of high-energy lithium- and manganese-rich layered oxides

Hua et al.

Supplementary Figures

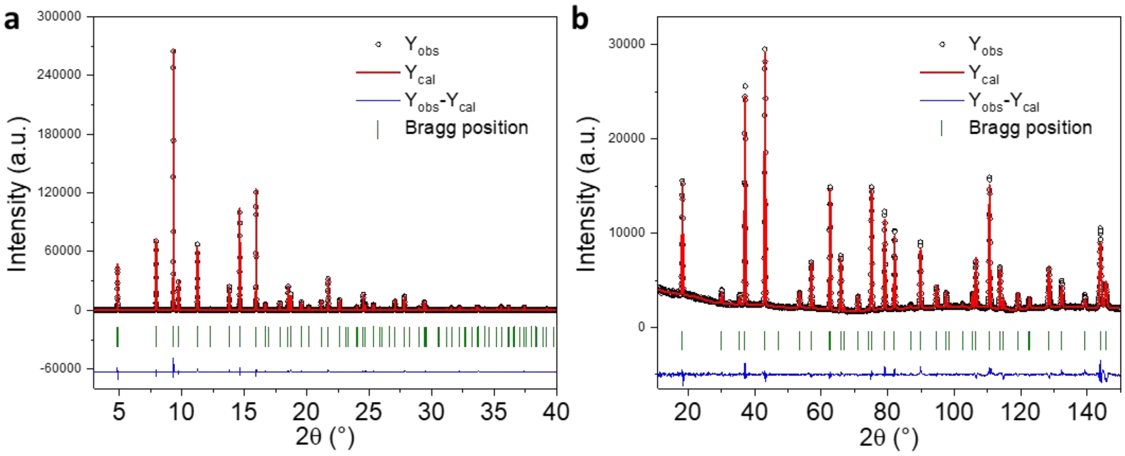

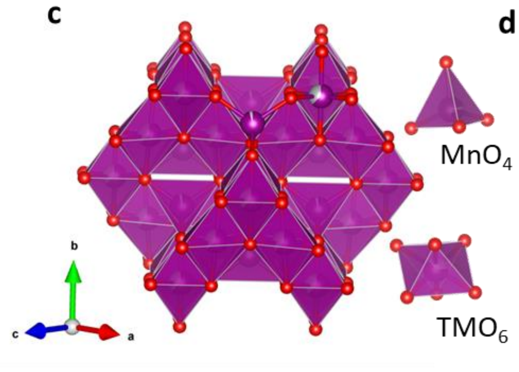

d

Cell parameters

Space group: *Fd-3m*, *a* = *b* = *c* = 8.4213 Å, *V* = 579.2285 Å^3

Atomic positions

Name	site	x	y	z	Fract
Mn1	*8a*	0.125	0.125	0.125	1.000
Ni1	*16d*	0.500	0.500	0.500	0.378
Mn2	*16d*	0.500	0.500	0.500	0.622
O1	*32e*	0.263	0.263	0.263	1.000

Refinement parameters

R_{wp}= 3.85 %, R_p= 2.95 % for NPD; R_{wp}= 10.00 %, R_p= 6.97 % for SRD

Supplementary Figure 1. Structural analysis of $Ni_{0.75}Mn_{2.25}O_4$ (L0.00). Simultaneous Rietveld refinement against **a** SRD (λ = 0.41231 Å) and **b** NPD (λ = 1.54825 Å) patterns of L0.00; **c** the obtained structure model and **d** crystallographic parameters of L0.00.

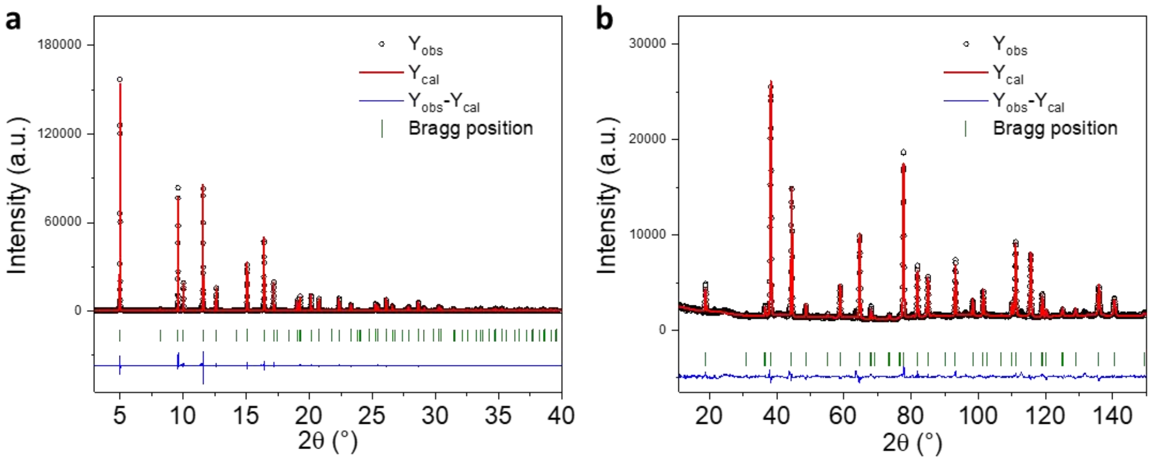

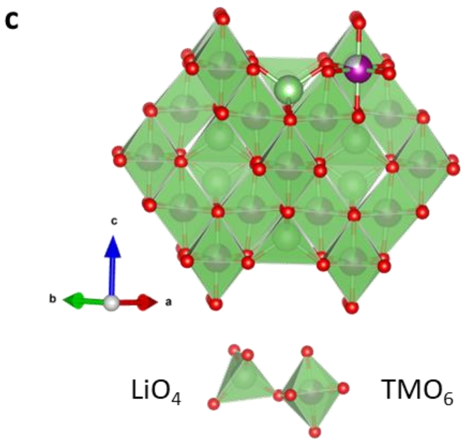

d

Cell parameters
Space group: *Fd-3m*, , *a* = *b* = *c* = 8.1843 Å, V = 548.2050 Å^3

Atomic positions

Name	site	x	y	z	Fract
Li1	*8a*	0.125	0.125	0.125	0.973
Ni1	*8a*	0.125	0.125	0.125	0.030
Li2	*16d*	0.500	0.500	0.500	0.015
Ni2	*16d*	0.500	0.500	0.500	0.235
Mn1	*16d*	0.500	0.500	0.500	0.750
O1	*32e*	0.263	0.263	0.263	1.000

Refinement parameters
R_{wp}= 4.18 %, R_p= 5.65 % for NPD; R_{wp}= 14.50 %, R_p= 9.52 % for SRD

Supplementary Figure 2. Structural analysis of $LiNi_{0.5}Mn_{1.5}O_4$ (L0.40). Simultaneous Rietveld refinement against **a** SRD (λ = 0.41231 Å) and **b** NPD (λ = 1.54825 Å) patterns of L0.40; **c** the obtained structure model and **d** crystallographic parameters of L0.40.

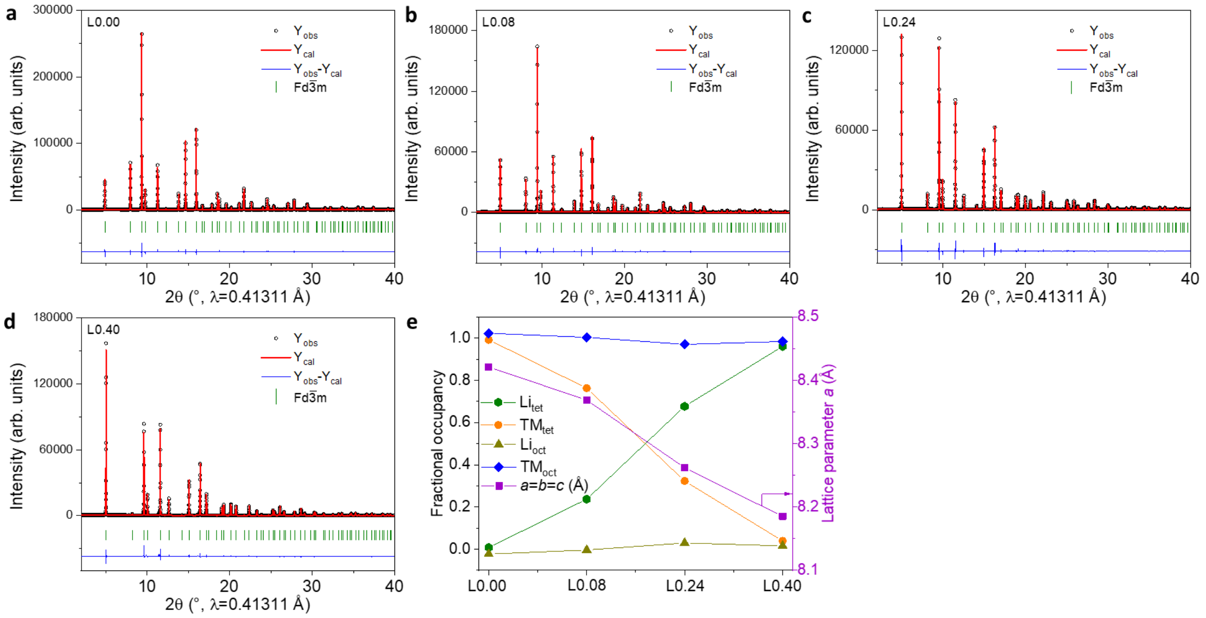

Supplementary Figure 3. Structural analysis of spinel oxides (L0.00 to L0.40). Rietveld refinement against synchrotron radiation diffraction patterns of **a** L0.00, **b** L0.08, **c** L0.24 and **d** L0.40 according to the structural model $Li_x\,Mn_{1-x_{8a,tet}}[Li_y\,Mn_{1-y}]_{16d,oct}(O_4\,)_{32e}$; **e** the obtained lattice parameters of the samples.

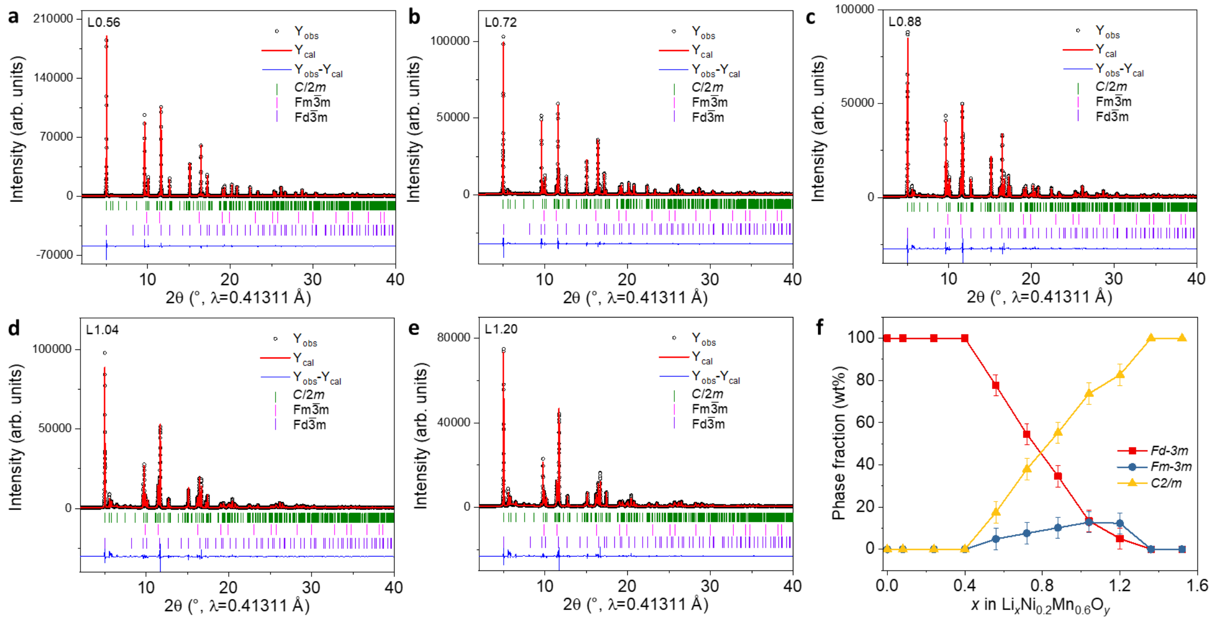

Supplementary Figure 4. Structural analysis of oxides with multiple phases (L0.40 to L1.20). Rietveld refinement against synchrotron radiation diffraction patterns of **a** L0.56, **b** L0.72, **c** L0.88, **d** L1.04 and **e** L1.20; **f** the resultant weight fraction of spinel/rock-salt-type/layered phases as a function of provided Li content.

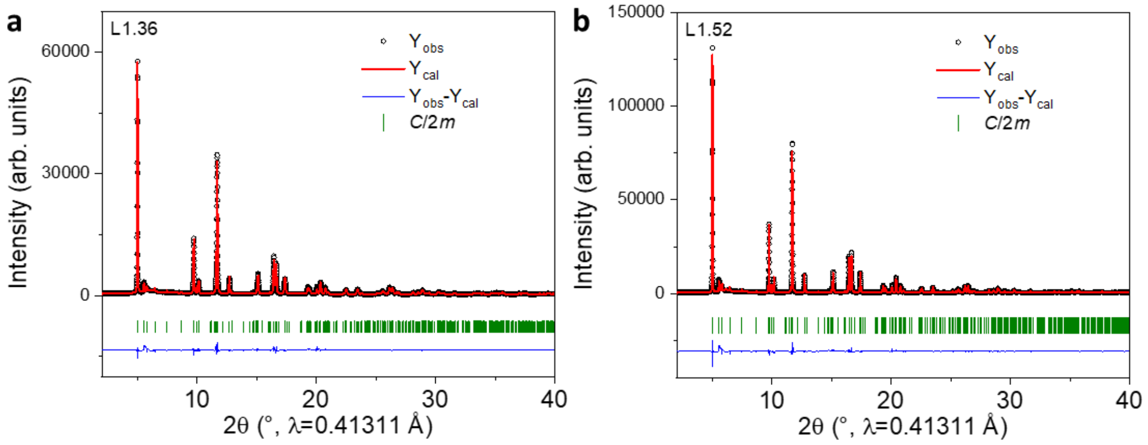

Supplementary Figure 5. Structural analysis of layered oxides (> L1.20). Rietveld refinement against synchrotron radiation diffraction patterns of **a** L1.36 and **b** L1.52.

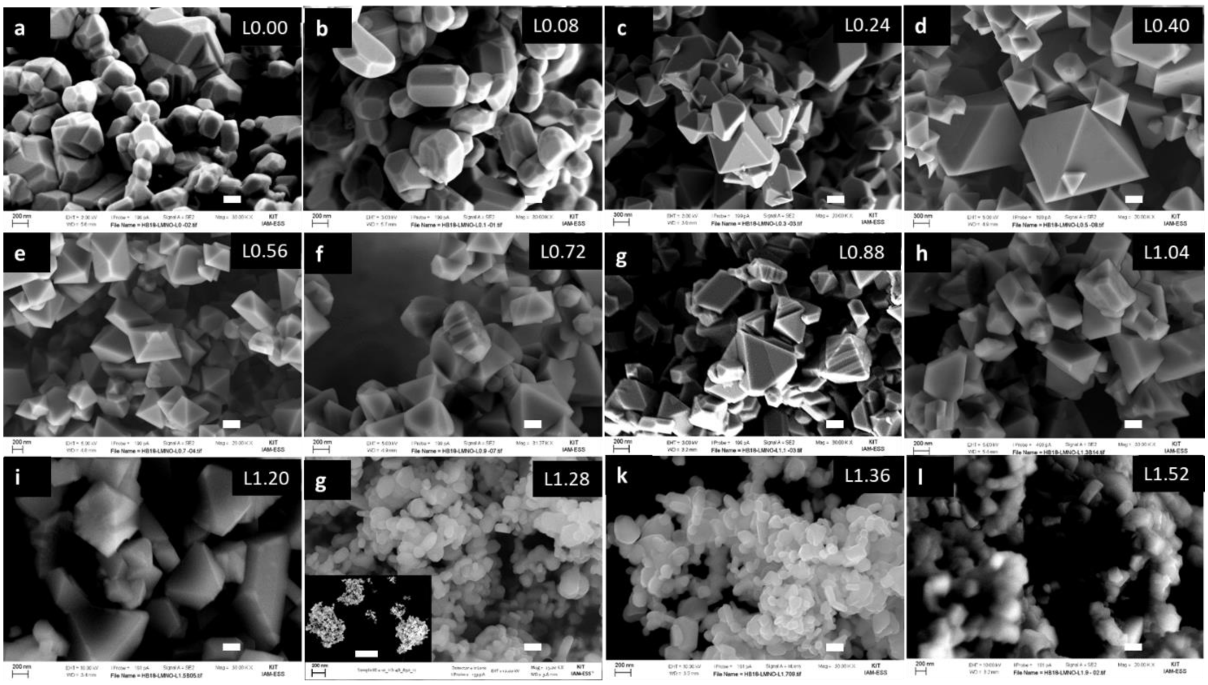

Supplementary Figure 6. SEM images of the samples obtained by adding different amounts of lithium. Scale bars: 200 nm (**a**–**b, e–l**), 300 nm (**c**–**d**), 10 μm (g, inset).

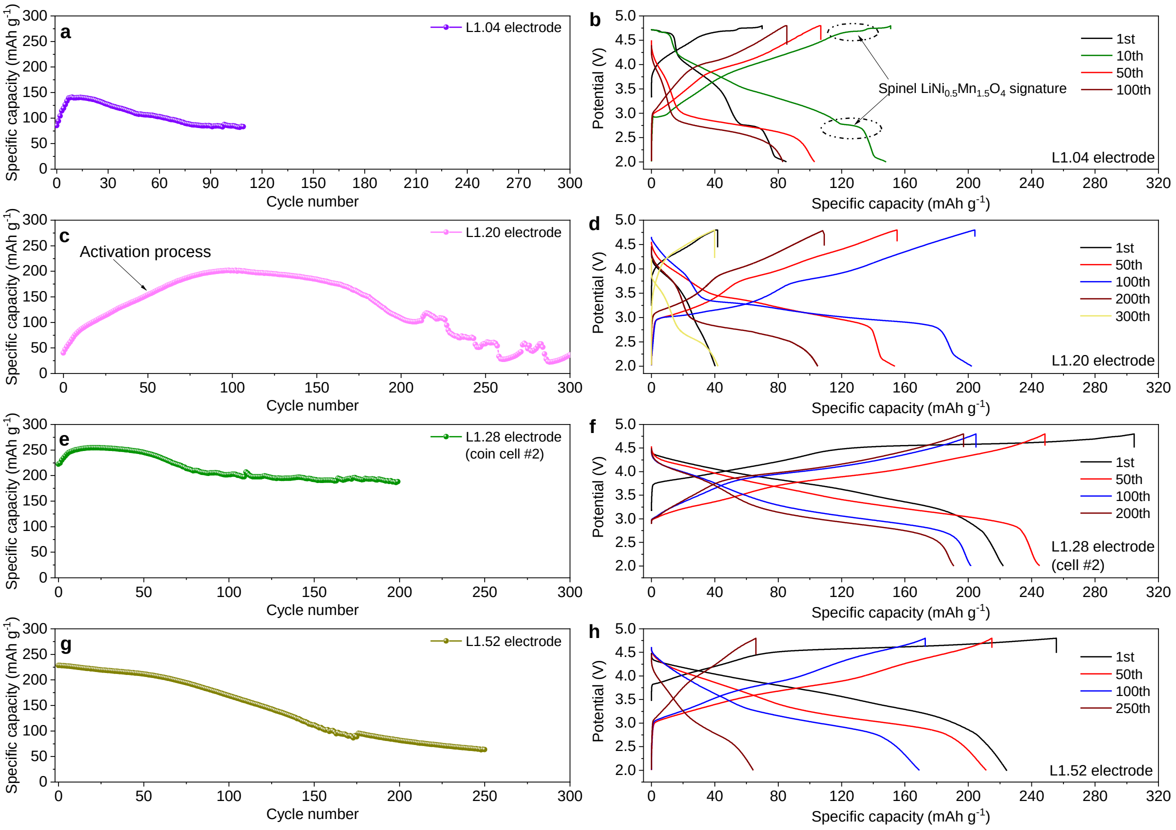

Supplementary Figure 7. Electrochemical properties of the selected electrodes. Galvanostatic cycling of **a** L1.04 electrode with spinel & rock-salt-type & layered phases, **c** L1.20 electrode with mainly layered rock-salt & Li-containing phases, **e** L1.28 electrode and **g** L1.52 electrode with monoclinic layered phase; the corresponding charge–discharge voltage profiles of the selected electrodes **b**, **d**, **f**, **h** between 2.0 and 4.8 V at a current density of 32 mA g^{-1} at room temperature.

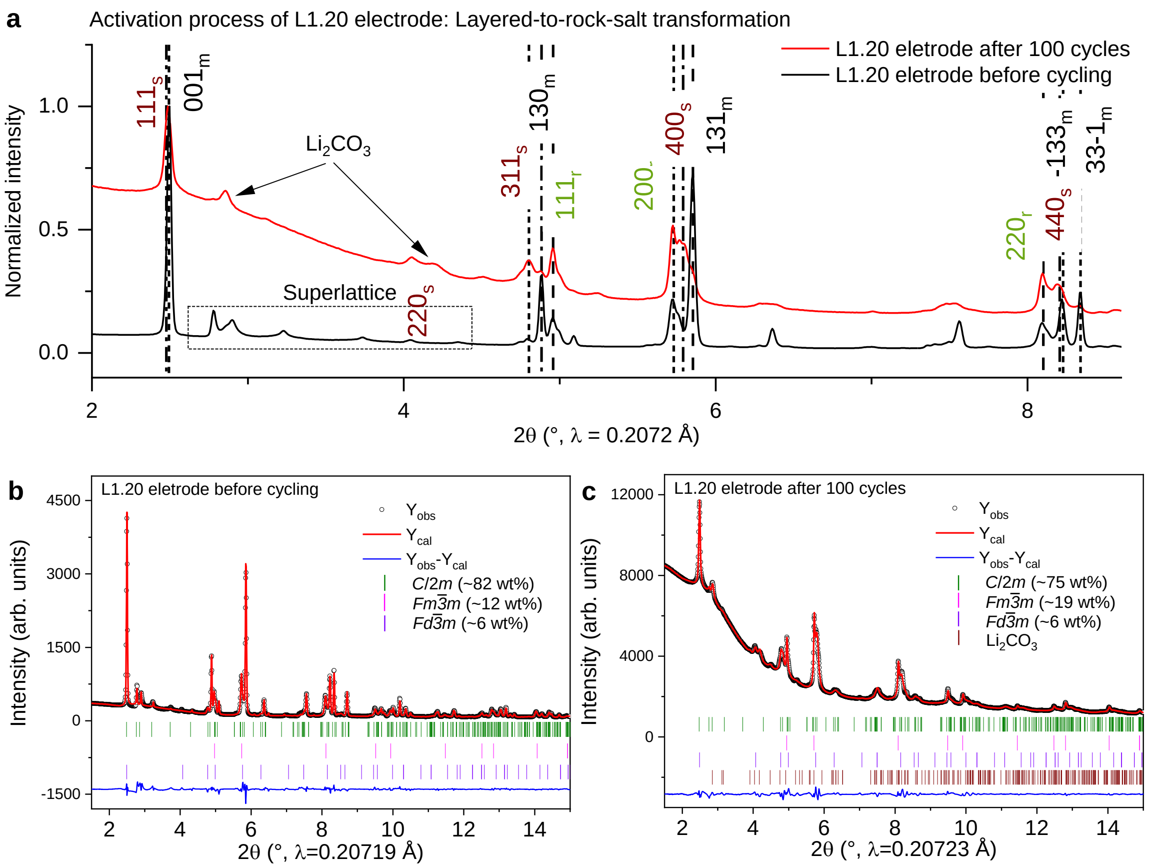

Supplementary Figure 8. Structural degradation of L1.20 cathode. Synchrotron radiation diffraction patterns of **a** L1.20 electrode before cycling and after 100 cycles; Rietveld refinement against synchrotron radiation diffraction patterns of L1.20 electrode **b** before cycling and **c** after 100 cycles. These results reveal that the phase transformation from ordered layered (*C2/m*) to defective/Li-concatining disordered rock-salt (*Fm-3m*) structure is responsible for the activation process of L1.20 electrode, rather than the formation of spinel (*Fd-3m*) phase from 1 to 100 cycles. Note that in order to show the composition changes of layered, rock-salt and spinel phases after the activation process, the phase fraction of Li_2CO_3 in the L1.20 electrode after 100 cycles was not accounted for in Figure **c**.

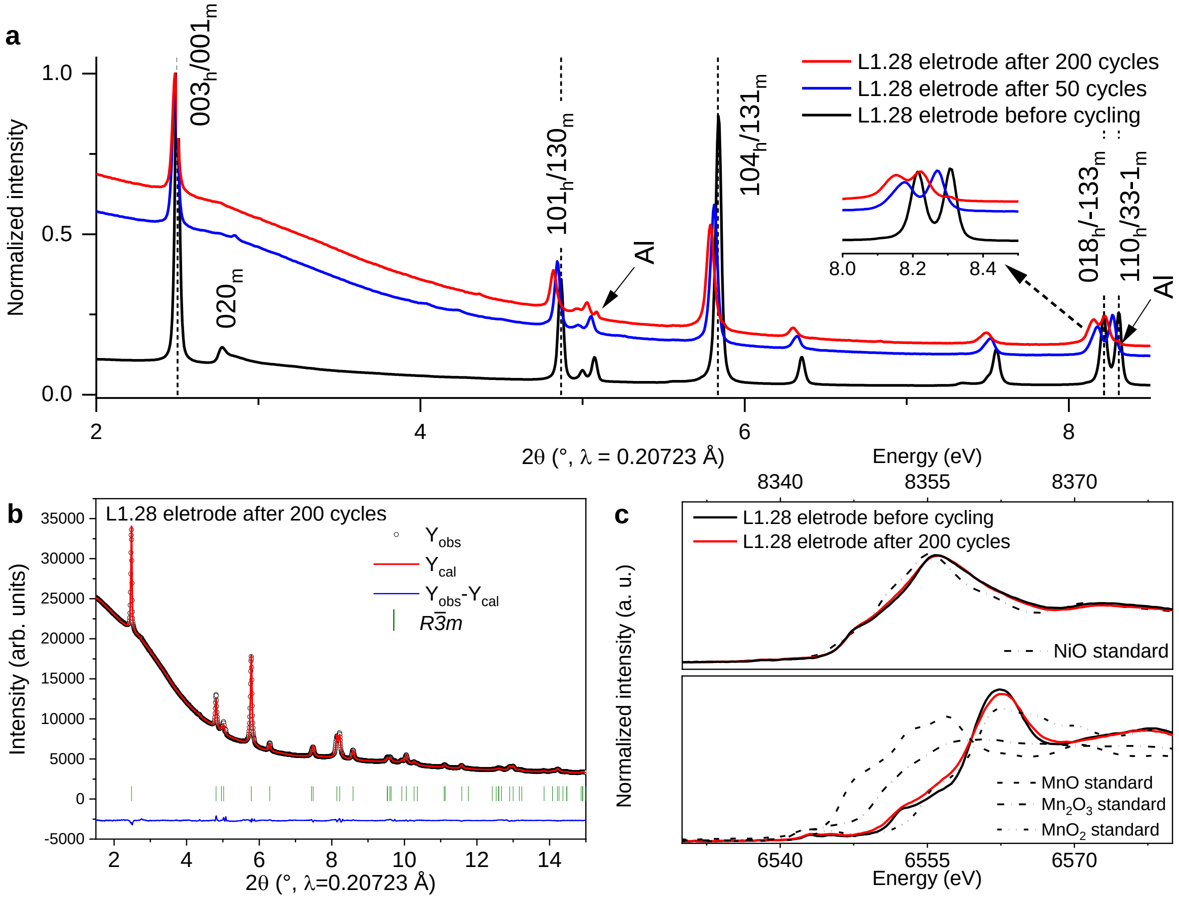

Supplementary Figure 9. Structural degradation of L1.28 cathode. Synchrotron radiation diffraction patterns of **a** L1.28 electrode before cycling and after 50, 200 cycles; **b** Rietveld refinement against synchrotron radiation diffraction patterns of L1.28 electrode after 100 cycles; **c** Ni K-edge and Mn K-edge XAS spectra of the L1.28 electrode before cycling and after 200 cycles.

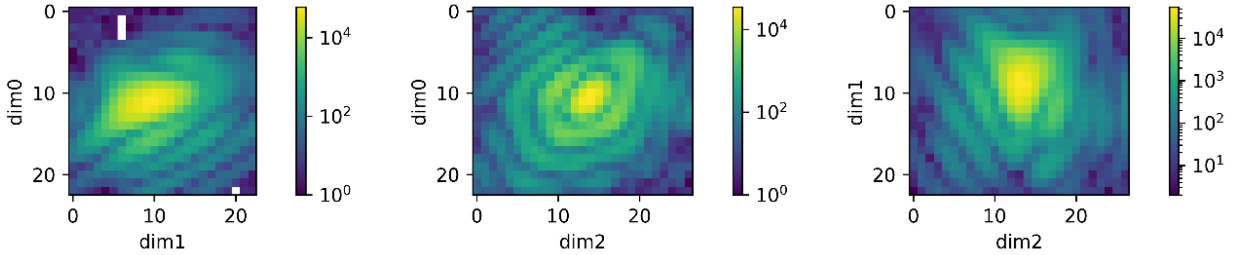

Supplementary Figure 10. 2D cross sections through the measured 3D 001_m Bragg peak intensity from a single crystallite demonstrates the oversampling ratio in each dimension is sufficient for phase retrieval. White pixels denote hot pixels, they are not included in the analysis as their value cannot be trusted.

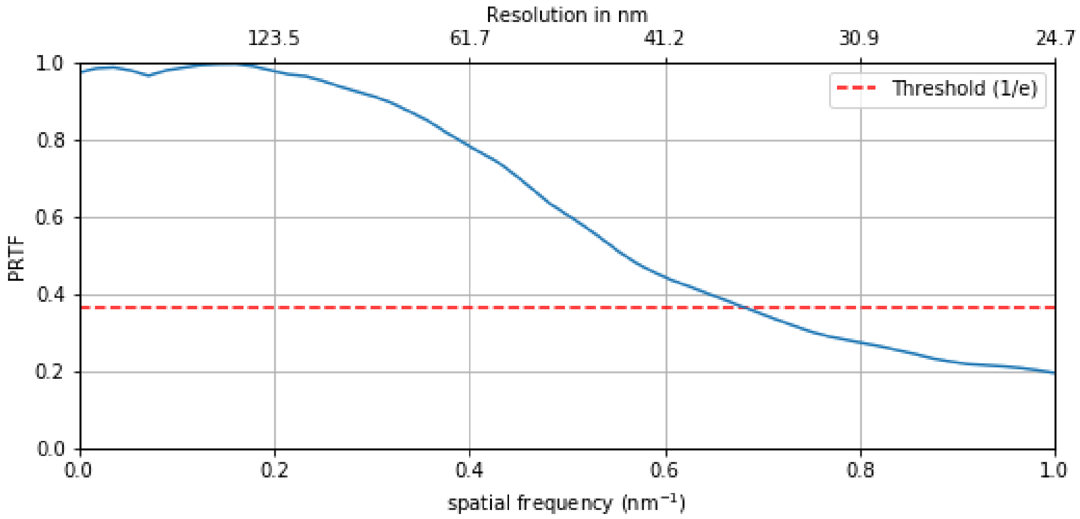

Supplementary Figure 11. Phase retrieval transfer function for the best reconstruction. The resolution on average in 3D is 35 nm.

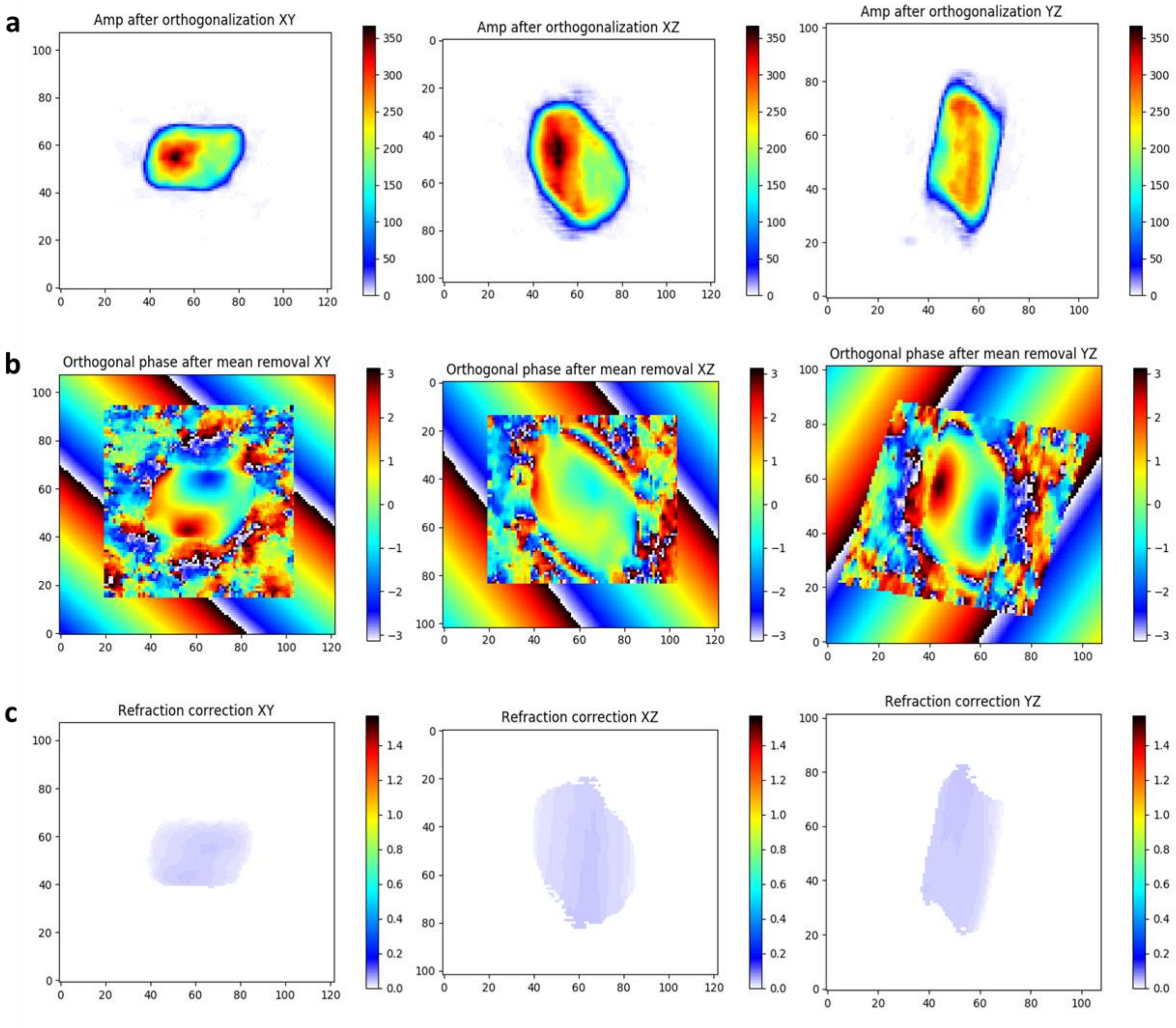

Supplementary Figure 12. BCDI reconstruction after phase ramp removal and corresponding refraction correction. 2D cross sections in the XY, XZ, YZ planes for the best eigen solution **a** amplitude (Bragg density), **b** phase (displacement field), and **c** refraction correction based on the optical path length.

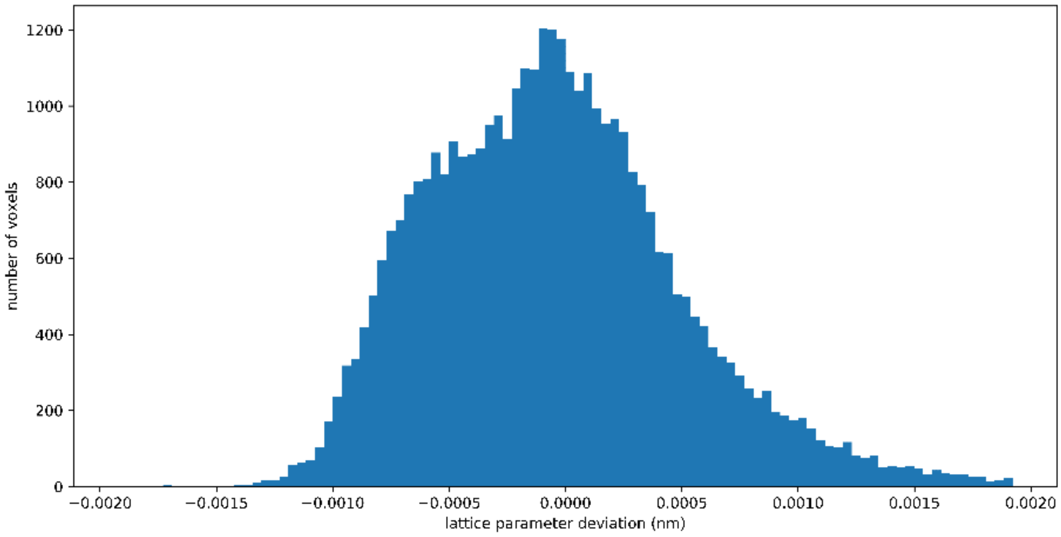

Supplementary Figure 13. Histogram of the lattice parameter distribution within the crystallite relative to 0.4805 nm.

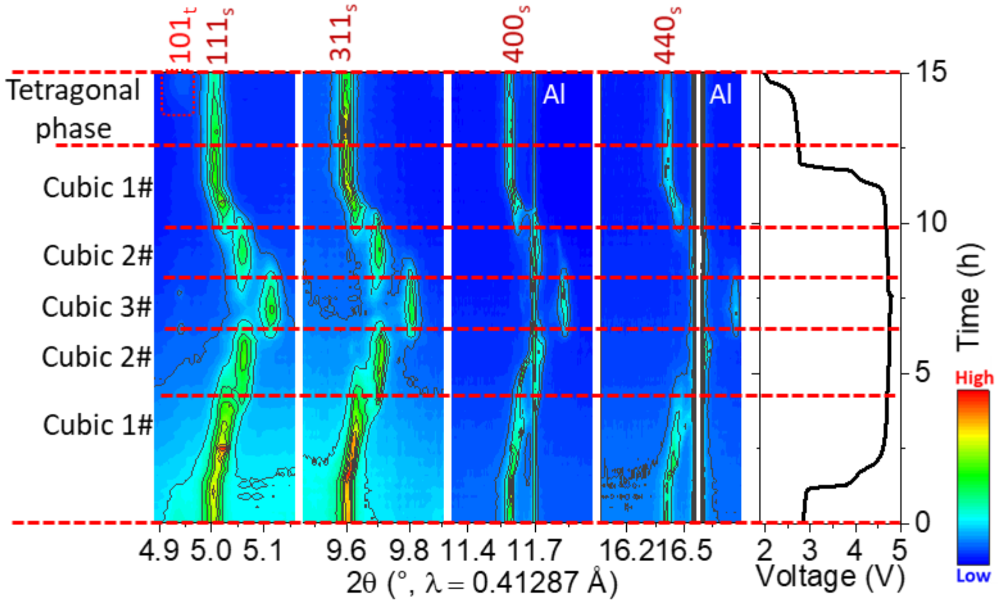

Supplementary Figure 14. *In situ* high-resolution SRD pattern of the spinel L0.40 electrode.

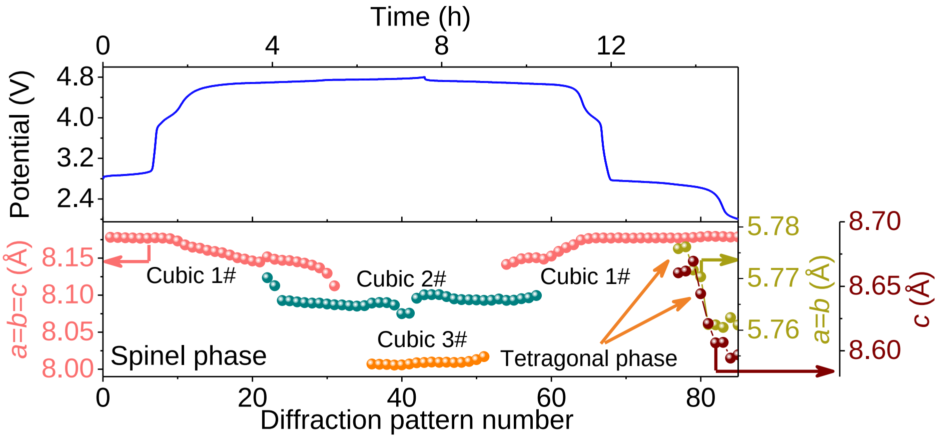

Supplementary Figure 15. Evolution of lattice parameters *versus* diffraction pattern number as obtained from Rietveld refinement against *in situ* synchrotron diffraction measurements of the L0.40 electrode.

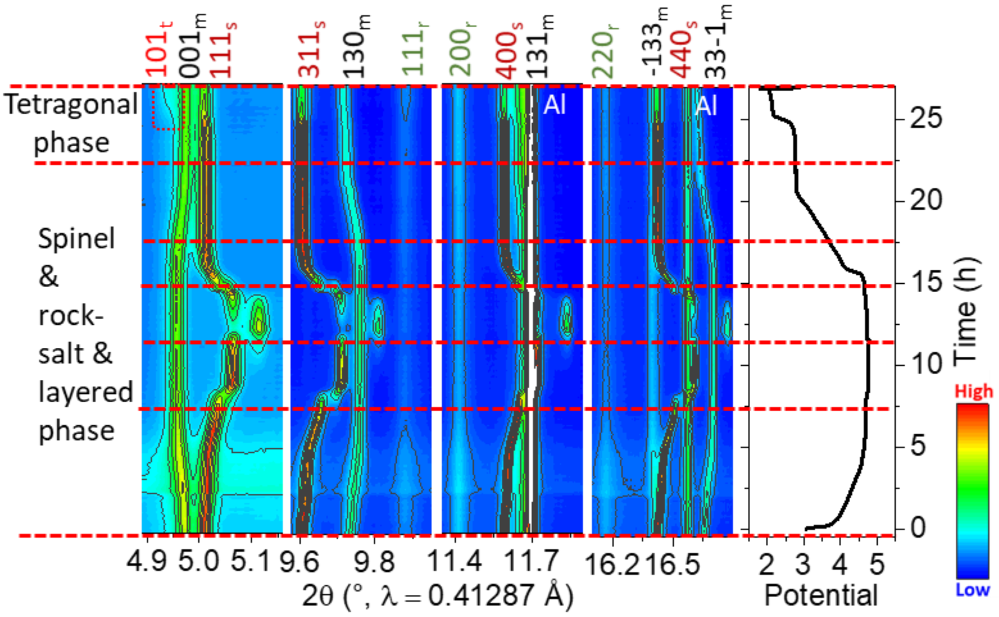

Supplementary Figure 16. *In situ* high-resolution SRD pattern of the multi-structured L0.88 cathode.

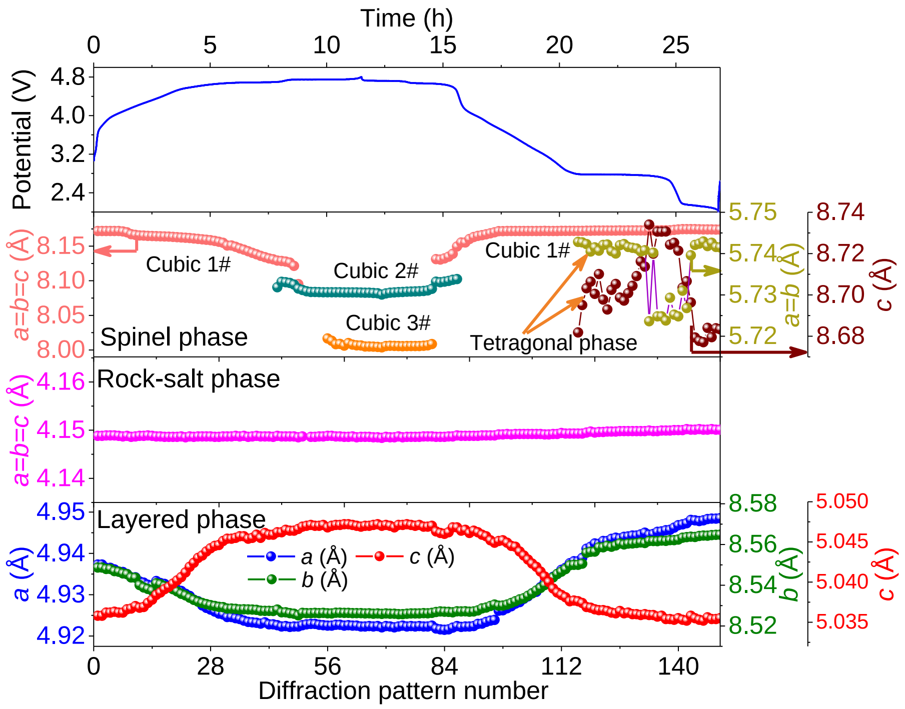

Supplementary Figure 17. Evolution of lattice parameters *versus* diffraction pattern number as obtained from Rietveld refinement against *in situ* synchrotron diffraction measurements of the L0.88 electrode.

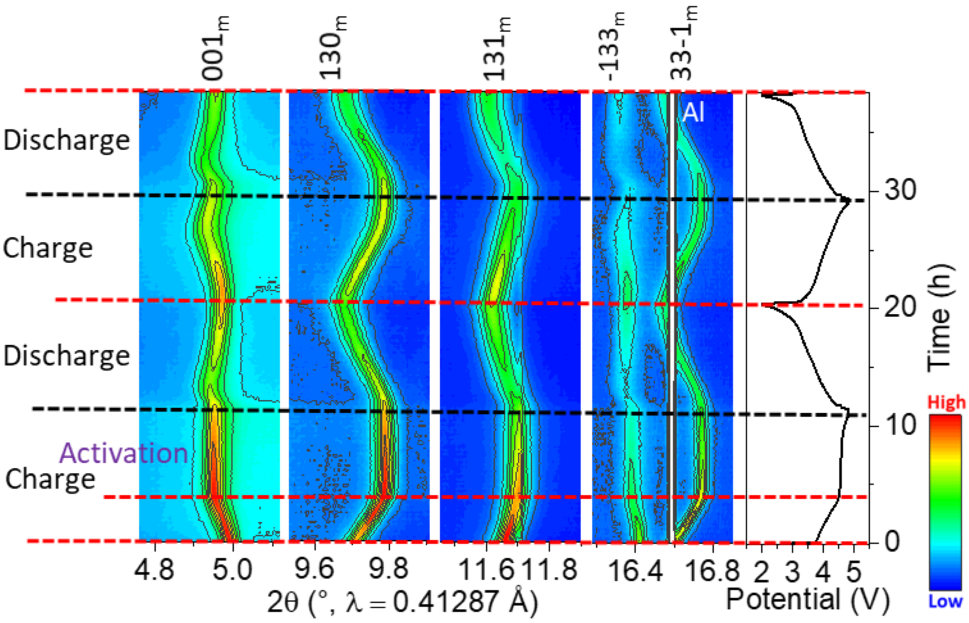

Supplementary Figure 18. *In situ* high-resolution SRD pattern of the monoclinic layered L1.28 cathode.

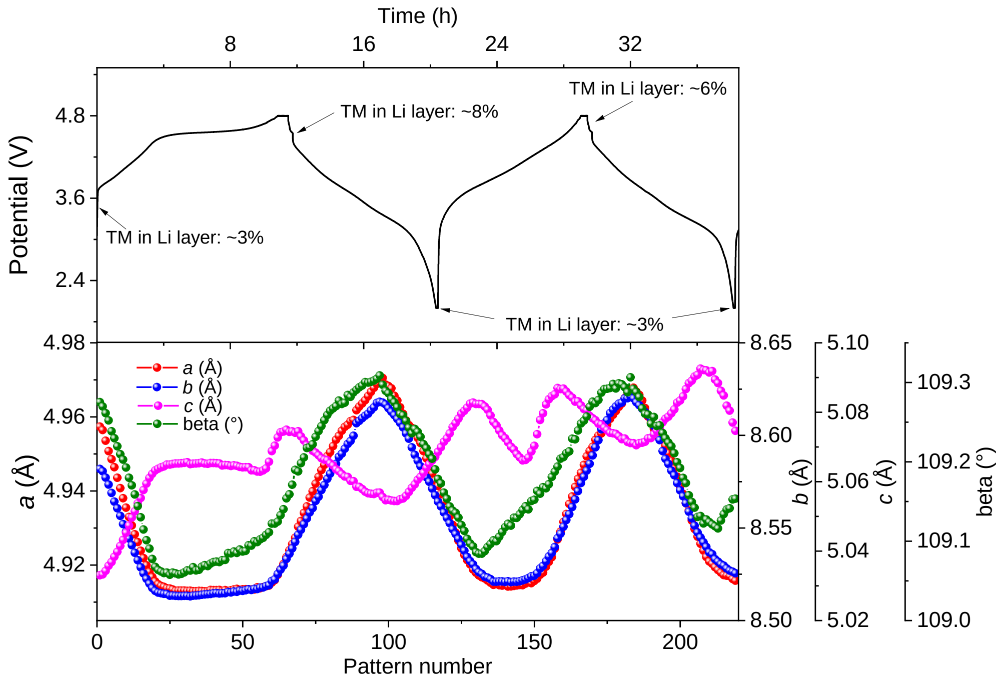

Supplementary Figure 19. Evolution of lattice parameters *versus* diffraction pattern number as obtained from Rietveld refinement against *in situ* synchrotron diffraction measurements of the L1.28 electrode.

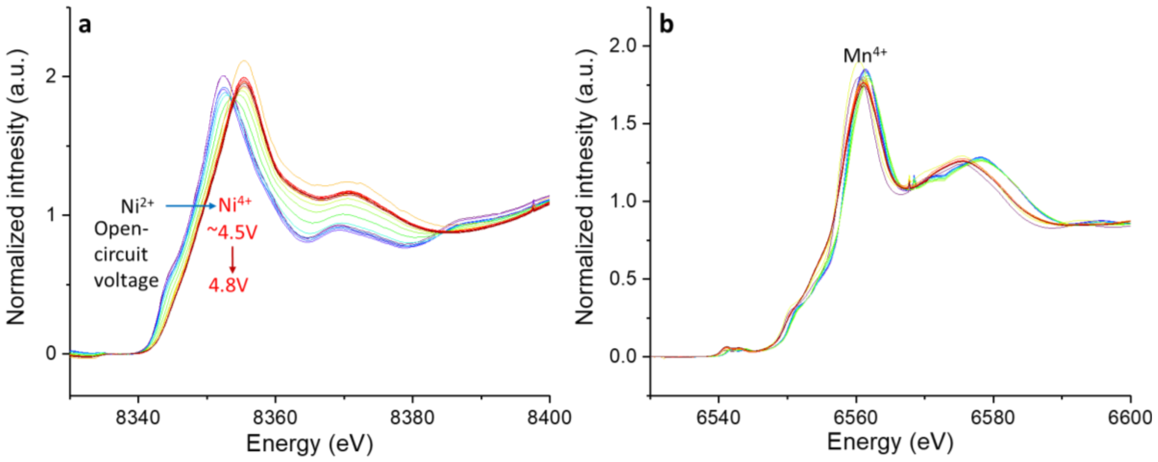

Supplementary Figure 20. Charge compensation mechanism in the L1.28 cathode. The normalized X-ray absorption near edge structure (XANES) spectra at **a** Ni K-edge and **b** Mn K-edge of the L1.28 electrode during the first charging process.

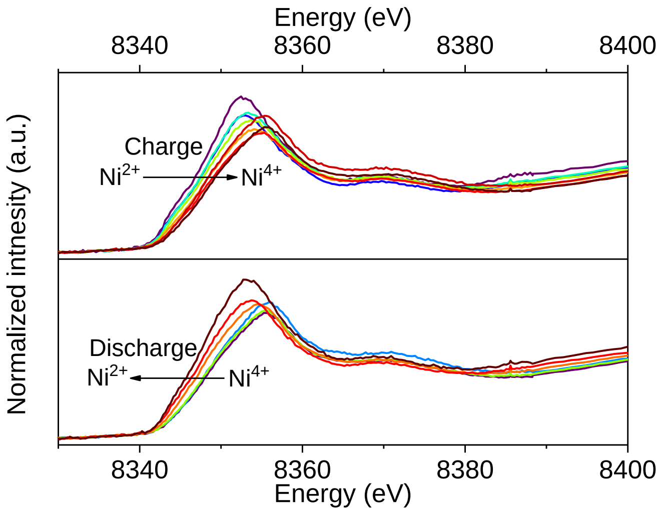

Supplementary Figure 21. The normalized XANES spectra at Ni K-edge of the L1.28 electrode during the 10th cycle.

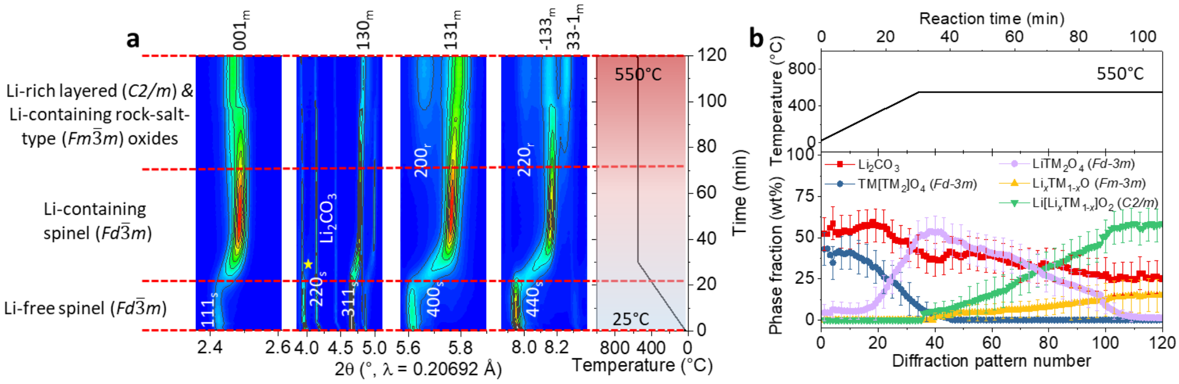

Supplementary Figure 22. Time-resolved high-temperature SRD patterns of a mixture of L0.00 and Li_2CO_3 during a slow thermal treatment.

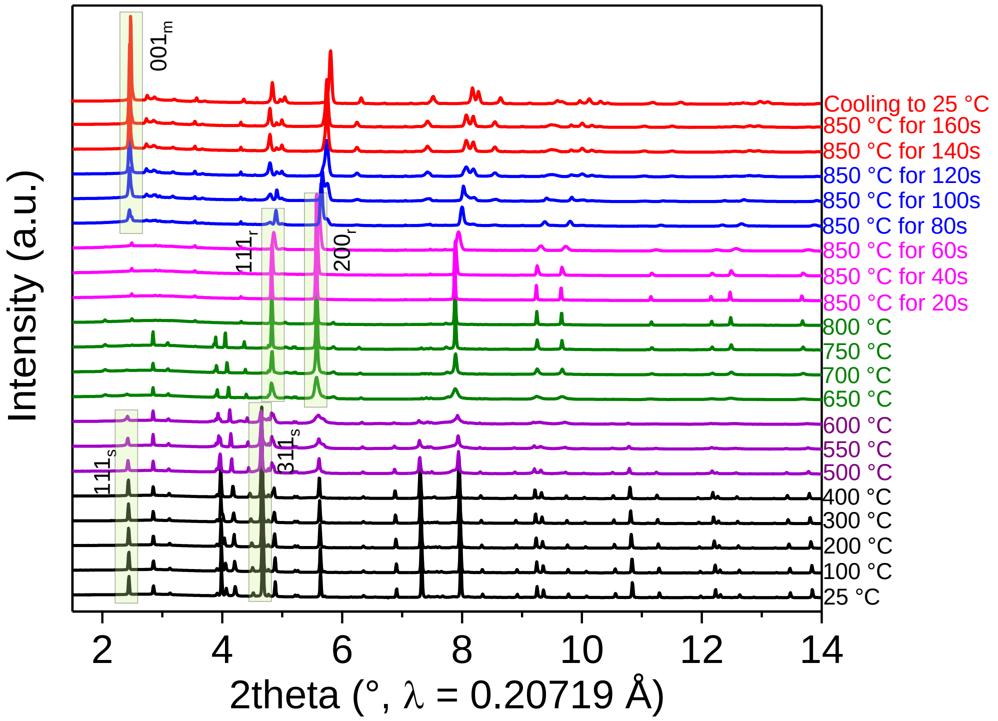

Supplementary Figure 23. Time-resolved high-temperature SRD patterns of a mixture of L0.00 and Li_2CO_3 during the fast thermal treatment process.

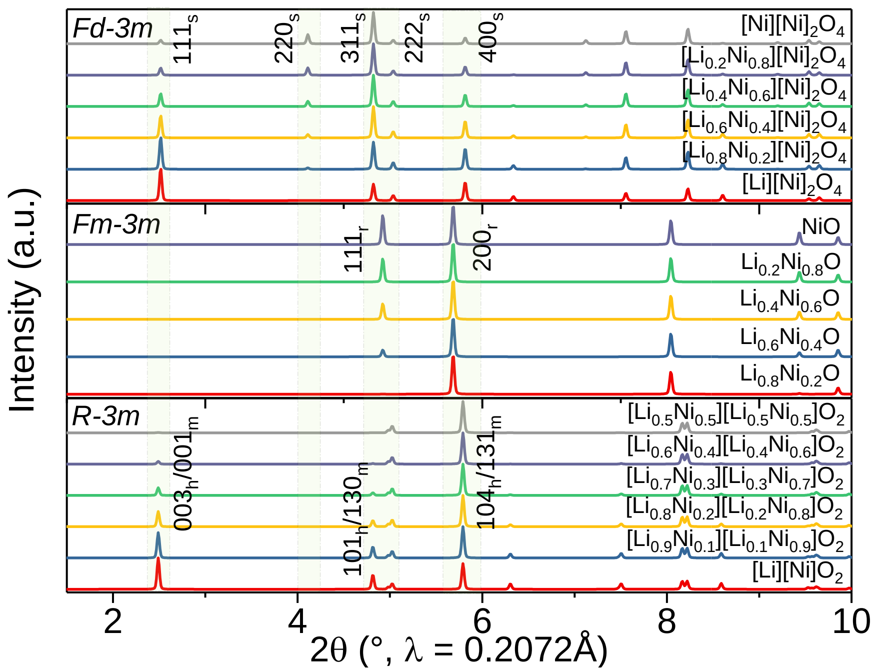

Supplementary Figure 24. Simulation results of X-ray powder diffraction patterns of spinel $[Li_xNi_{1-x}][Ni_2]O_4$, rock-salt $[Li_xNi_{1-x}]O$ phase, and layered hexagonal $[Li_xNi_{1-x}][Ni_xLi_{1-x}]O_2$. s, r, h and m represent spinel, rock-salt, hexagonal and monoclinic layered phase, respectively.

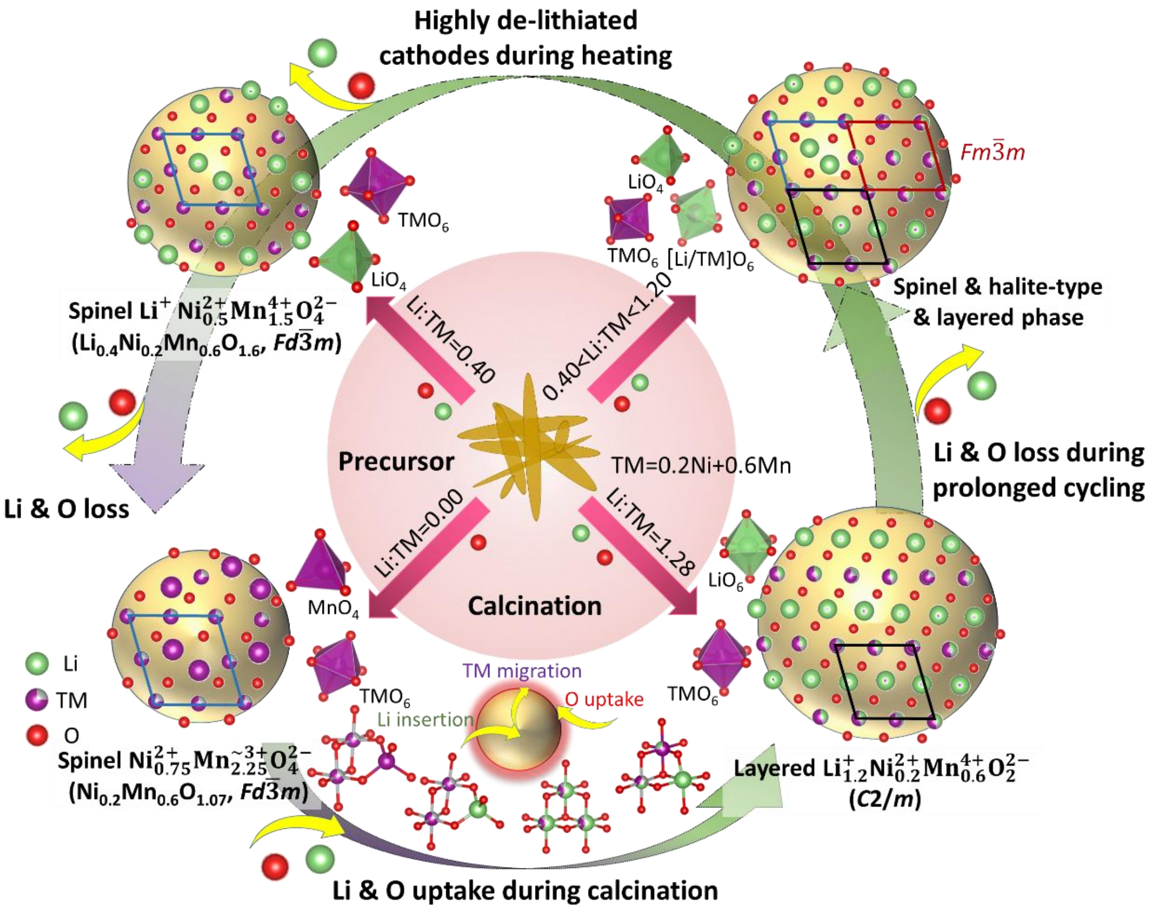

Supplementary Figure 25. Schematic diagram of lithium- and oxygen-driven structural, electrical and thermodynamic complexity in Li-Ni-Mn-O systems, revealing the tendency of the Mn-rich oxides towards formation of different phases, depending on the Li content in the system, during synthesis and discharge-charge process of LMLOs.

Supplementary Tables

Supplementary Table 1. Results of chemical analysis of the samples.

Samples	Molar ratio			
	Li	Ni	Mn	O
L1.28	1.24	0.2	0.55	1.95
L0.40	0.36	0.2	0.55	1.48

Standard deviation: < 2 %.

Supplementary Table 2. Lattice parameters and structural parameters of the samples with provided different lithium contents.

Sample	Layered phase ($C2/m$)					Spinel phase ($Fd\overline{3}m$)		Rock-salt phase ($Fm\overline{3}m$)	
	a (Å)	b (Å)	c (Å)	beta	fraction (wt.%)	a (Å)	fraction (wt.%)	a (Å)	fraction (wt.%)
L0.00	/	/	/	/	/	8.4211	100 (0.49)	/	/
L0.08	/	/	/	/	/	8.3687	100 (0.32)	/	/
L0.24	/	/	/	/	/	8.2620	100 (0.36)	/	/
L0.40	/	/	/	/	/	8.1852	100 (0.64)	/	/
L0.56	4.9354	8.5449	5.0263	108.916	17.51 (0.27)	8.1712	77.63 (0.39)	4.1454	4.86 (0.19)
L0.72	4.9342	8.5459	5.0271	108.926	37.98 (0.42)	8.1705	54.47 (0.37)	4.1469	7.55 (0.26)
L0.88	4.9343	8.5464	5.0274	108.930	55.25 (0.58)	8.1693	34.57 (0.33)	4.1470	10.18 (0.34)
L1.04	4.9343	8.5465	5.0273	108.929	73.79 (0.78)	8.1693	13.41 (0.24)	4.1470	12.80 (0.42)
L1.20	4.9343	8.5464	5.0274	108.930	82.69 (0.84)	8.1693	5.04 (0.16)	4.1470	12.27 (0.46)
L1.36	4.9611	8.5651	5.0314	109.266	100 (0.55)	/	/	/	/
L1.52	4.9423	8.5541	5.0254	109.267	100 (0.56)	/	/	/	/

Supplementary Table 3. Crystallographic parameters of L1.28.

Cell parameters						
Space group: *C2/m*, a = 4.9585 Å, b = 8.5804 Å, c = 5.0332 Å , V = 202.1403 Å^3, β = 109.27°						
Atomic positions						
Name	site	*x*	*y*	*z*	B_{iso}	Fract
Li1	2*c*	0.000	0.000	0.500	0.559	1.000
Li2	4*h*	0.000	0.694	0.500	0.559	0.944
Ni2	4*h*	0.000	0.694	0.500	0.559	0.054
Ni1	2*b*	0.000	0.500	0.000	0.559	0.212
Li3	2*b*	0.000	0.500	0.000	0.559	0.416
Mn2	2*b*	0.000	0.500	0.000	0.559	0.380
Mn1	4*g*	0.000	0.170	0.000	0.559	0.710
Ni3	4*g*	0.000	0.170	0.000	0.559	0.140
Li4	4*g*	0.000	0.170	0.000	0.559	0.149
O1	4*i*	0.224	0.000	0.224	0.347	1.000
O2	8*j*	0.256	0.329	0.2276	0.933	1.000
Refinement parameters						
R_{wp}= 4.58 %, R_p= 3.62 %, χ^2: 1.39 for neutron powder diffraction						
R_{wp}= 6.39 %, R_p= 4.36 %, χ^2: 4.05 for synchrotron radiation powder diffraction						

Note: B_{iso} is the thermal displacement parameter, Fract is the fractional occupancy of the atom on this site.

Supplementary Table 4. Lattice parameters of the 1.28 electrode before cycling and after different cycles.

L1.28 eletrode	Structural mode (*R-3m*)						
	a (Å)	*c* (Å)	*c/a*	*V* (Å^3)	I_{003}/I_{104}	TM in the Li layer (%)	Average maximum strain ($\Delta d/d$ 10^{-4})
Before cycling	2.8614	14.2575	4.9827	101.98	0.88	2.80	20.38
After 50 cycles	2.8615	14.2573	4.9824	101.10	0.89	2.50	29.17
After 200 cycles	2.8752	14.3373	4.9862	102.64	0.76	5.60	42.43
After 868 cycles	2.9122	14.9827	4.9387	105.635	0.61	5.90	107.58

Supplementary Table 5. Phase constitution of the mixture of L0.00 and Li_2CO_3 at different temperatures.

SRD pattern at different temperatures	Phase fraction (wt.%)						
	Li_2CO_3 ($C2/m$)	$Li_xTM_{1-x}TM_2O_4$ ($I4_1$/amd)	$Li_xTM_{1-x}TM_2O_4$ ($Fd\bar{3}m$)	$Li[Li_xTM_{1-x}]O_2$ ($C2/m$)	Li_2TMO_2 ($P3m1$)	$Li_xTM_{1-x}O$ ($Fm\bar{3}m$)	I_{111r}/I_{200r}
25 °C	48.26 (0.90)	/	51.74 (0.49)	/	/	/	
100 °C	49.04 (0.91)	/	50.96 (0.48)	/	/	/	
200 °C	50.80 (0.94)	/	49.20(0.48)	/	/	/	
300 °C	48.15 (0.98)	/	51.85 (0.52)	/	/	/	
400 °C	50.76 (1.04)	/	49.24 (0.53)	/	/	/	
500 °C	52.88 (1.39)	/	35.79 (0.61)	/	/	11.33 (0.96)	0.68
550 °C	53.46 (1.68)	/	29.68 (0.76)	/	/	16.87 (0.93)	0.74
600 °C	46.45 (1.51)	11.80 (0.90)	10.23 (0.47)	/	/	31.51 (1.42)	0.46
650 °C	32.38 (0.85)	8.80 (0.92)	/	/	5.06 (0.67)	53.76 (1.54)	0.43
700 °C	32.29 (0.83)	4.72 (0.43)	/	/	4.25 (1.24)	58.74 (1.38)	0.45
750 °C	28.97 (1.20)	2.54 (0.25)	/	/	3.28 (0.42)	65.21 (0.84)	0.39
800 °C	/	/	/	3.37 (0.05)	2.93 (0.43)	89.42 (1.77)	0.36
850 °C for 20s	/	/	/	0.18 (0.19)	/	93.53 (1.27)	0.35
850 °C for 40s	/	/	/	0.19 (0.23)	/	92.80 (1.57)	0.34
850 °C for 60s	/	/	/	0.74 (0.19)	/	91.72 (1.33)	0.30
850 °C for 80s	/	/	/	28.08 (0.69)	/	65.08 (1.13)	0.28
850 °C for 100s	/	/	/	56.50 (1.04)	/	37.13 (0.93)	0.29
850 °C for 120s	/	/	4.06 (0.55)	82.92 (1.44)	/	4.72(0.25)	0.31
850 °C for 140s	/	/	2.76 (0.38)	85.97 (1.19)	/	2.71 (0.23)	
850 °C for 160s	/	/	/	90.88 (1.00)	/	/	

Note: the impurity of Li_2SiO_3 in the sample obtained at above 800 °C was not accounted in this table.

Supplementary Notes

Supplementary Figure 6 shows the morphological changes of the samples with increasing lithium concentration. Concerning the L0.00, the particles with spinel structure have a round, polyhedral shape. When the Li content is increasd to L0.40, the size of the primary particles increases and the particle shape changes to an octahedral shape with pronounced edges and corners. From L0.40 to L1.20, the fine crystals tend to form the plate-like morphology. When the Li concentration is further increased to L1.52, the primary particle size of the crystals is decreased, the plate-like particles convert to a spherical or polyhedral shape. These results provide evidence of surface reconstruction to accommodate more lithium and oxygen atoms during thermal treatment.

Supplementary Figure 7 shows the electrochemical performance and selected charge–discharge voltage profiles of four typical samples, i.e. L1.04, L1.20, L1.28 and L1.52. When compared with the layered L1.28 and L1.52 cathodes, both L1.04 and L1.20 electrodes with layered and spinel/rock-salt-type phases exhibit a poor cycling performance and a severe voltage decay upon cycling. Regarding the L1.20 electrode, after 100 cycles at charge/discharge rate of 0.1 C, nearly 500 % of the initial discharge capacity can be achieved, indicating a continuous electrochemically induced activation process of Li-containing/defective disordered rock-salt phase during cycling, see **Supplementary Figure 8**. Note that the L1.20 electrode experiences a rapid capacity loss after activation process (i.e. after 100 cycles), which probably results from the decomposition of active materials, mostly Mn^{3+} in the spinel phase (see the plateau at ~ 2.7 V), after the formation of Li-poor spinel phase caused by successive loss of Li/O. Obviously, the L1.52 cathode also suffers from a serious voltage fading during cycling, but the plateau of L1.52 cathode at about 2.7 V corresponding to the characteristics of spinel phase is much smaller than that of L0.88 and

L1.20 electrodes, suggesting that the voltage degradation of Li-rich layered cathodes is closely tied to the formation of Li-containing/defective rock-salt phase. Keep in mind that the oxidation state of nickel in the L1.52 electrode is higher than 2+, see **Figure 1c**, its capacity, thereby, mostly originates from oxygen redox during cycling. The oxygen anions can be easily removed from the layered structure of the L1.52 electrode when a large number of electrons is released from oxygen *2p* orbitals, thus resulting in a phase transition from layered to rock-salt and then to spinel structure, and consequently a poor electrochemical performance. Therefore, the electrochemical properties are strongly related to the phase and chemical composition of the cathode materials.

In situ SRD technique was employed to understand the relationship between structure and electrochemical property of L0.40 with a cubic spinel phase, L0.88 with a multiple-phase and L1.28 with a monoclinic layered phase during the first cycle. The evolution of structure and crystallographic parameter of these three samples is presented in **Supplementary Figures 14–19**.

For spinel phase L0.40, all the reflections shift to higher (lower) scattering angles as Li-ions are extracted from (inserted into) the host spinel structure. Three cubic phases are directly detected at about 4.7 V and these phases convert from one into another by way of two distinct two-phase regions that correspond to two wide potential plateaus observed in the electrochemical curves. These results agree well with the subtle changes in the lattice parameters of three cubic unit cells (**Supplementary Figure 14**). When more lithium ions are gradually intercalated into the unoccupied 16*c* octahedral site in the spinel structure, the tetragonal phase ($I4_1/\mathrm{amd}$) is formed at the discharge potential of around 2.7 V as a result of

the Li displacement from the LiO_4 tetrahedron (8*a* site) to the face-shared LiO_6 octahedron (16*c* site) [1].

Regarding the L0.88 electrode, the reflections representing the Li-containing rock-salt phase do not change substantially, as evidenced by the nearly unchanging lattice parameter of rock-salt unit cell during charge and discharge process (**Supplementary Figures 16–17**), suggesting that TM and Li cations in the rock-salt structure are electrochemically inactive. Encouragingly, except for the observation of typical spinel (de)lithiation characteristics, the structural evolution of the layered phase is also pronouncedly observed in the L0.88 electrode. According to the crystallography theory, 001_m and $\bar{1}33_m$ reflections sensitively reflect the changes along *c* axis, while the 130_m and $33\bar{1}_m$ reflections are indicative of the evolution along *a* and *b* axis of the monoclinic layered structure. As the charging potential increases to around 4.5 V, the 001_m reflection shifts to lower 2-theta angles and the 130_m reflection shifts to higher scattering angles with partially de-intercalating Li ions from the layered structure. Correspondingly, *c*-lattice parameter of layered unit cell is increased and lattice parameters of *a* and *b* are decreased, respectively, displaying an expansion electrostatic repulsion force among the ccp oxygen layers and a contraction TM-O bands in TMO_6 octahedra. When the L0.88 electrode is charged to a higher voltage (i.e. from 4.5 to 4.8 V), only typical cubic phase transformation is discovered while the lattice parameters of the layered structure are almost constant (vice versa on the discharge process).

With respect to the layered L1.28 cathode, during de-lithiation (charge), the 001_m and $\bar{1}33_m$ reflections continuously shift to lower scattering angles up to the voltage of 4.5 V and slightly back to higher angles after charging above 4.5 V (see **Supplementary Figure 18**), revealing that the *c*-lattice parameter is relatively enlarging and compressing upon the loss of the Li ions. At the same time, the 130_m and $33\bar{1}_m$ reflections shift to higher scattering angles as the

potential increases from the open-circuit voltage to 4.5 V and remain nearly constant in the voltage window of 4.5–4.8 V. The results indicate that the *a* and *b* lattice parameters decrease smoothly below 4.5 V and remain almost the same beyond 4.5 V, which can be attributed to the removal of electrons from Ni ions ($Ni^{2+} \rightarrow Ni^{4+} + 2e^{-}$) and from oxygen anions, respectively, see **Supplementary Figures 20–21**. This is in good agreement with a smooth and monotonous charging feature within the low voltage window and a pronounced voltage plateau (electrochemical activation process) at about 4.6 V, respectively. During lithiation (discharge) period, all the reflections tend to move back to their initial position signifying the evolution of lattice parameters in the reverse direction. Notably, the deviation of lattice parameters of the unit layered cell between the beginning and the end of the first cycle is not ignorable, see **Supplementary Figure 19**, this discrepancy can be ascribed to the irreversible loss of Li and/or oxygen after activation process and thus resulting in a low coulombic efficiency of the L1.28 electrode (~ 78 %) in the first cycle. During the second charging process, the 001_m reflection gradually shifts to lower scattering angles and then back to higher angles, revealing the expansion and contraction of the *c*-lattice parameter upon the loss of the lithium ion. At the same time, the 130_m reflection shifts to higher scattering angles, demonstrating the reduction of the *a* and *b* lattice constant (vice versa on the 2nd charging process).[2,3]

In situ XAS was used to trace the changes of the bulk oxidation states of the L1.28 electrode. During initial charge process, the *d* electrons of Ni^{2+} are gradually removed as the potential increases to approximately 4.5 V, while the oxidation state of Ni and Mn does not change dramatically (both are assigned to +4, see **Supplementary Figure 20**). These reveal that tetravalent Mn in octahedral coordination of Li-rich oxides cannot provide extra electrons. Since Ni^{2+} ($t_{2g}^{6}e_{g}^{2}$) / Ni^{4+} ($t_{2g}^{6}e_{g}^{0}$) redox can only compensate for 0.4 Li ion extraction from $Li_{1.2}Ni_{0.2}Mn_{0.6}O_2$, corresponding to a capacity of around 100 mA h g^{-1}, the high initial charge

capacity of L1.28 (~ 320 mA h g^{-1}) is supposed to be correlated to the electron loss from oxygen.

Supplementary Methods

Materials synthesis

The Mn-rich ($Li_xNi_{0.2}Mn_{0.6}O_y$, $0.00 \leq x \leq 1.52$) oxides were prepared by the following synthesis process,

$$\underbrace{0.2\mathrm{Ni}^{2+} + 0.6\mathrm{Mn}^{2+} + 2\mathrm{OH}^- \xrightarrow{\mathrm{NH_3{\cdot}H_2O}} \xrightarrow{\mathrm{air-drying}} \mathrm{precursor}}_{\mathrm{hydroxide\ co-precipitation\ reaction}}$$

$$\underbrace{\mathrm{precursor} + \frac{x}{2}\mathrm{Li_2CO_3} \xrightarrow{850\ ^\circ\mathrm{C,12h,air}} \mathrm{Li}_x\ \mathrm{Ni_{0.2}Mn_{0.6}O}_y}_{\mathrm{high-temperature\ lithiation\ reaction}}$$

$(0.00 \leq x \leq 1.52)$

Firstly, a 2 M aqueous solution of stoichiometrically mixed $NiSO_4{\cdot}6H_2O$ and $MnSO_4{\cdot}H_2O$ was pumped into a reactor at the adding rate of 2 ml min^{-1}. Simutaneously, 4 M NaOH solution and a proper amount of $NH_3{\cdot}H_2O$ solution was added into the reactor. The pH value (11.3 ± 0.3), temperature (52 ± 2 ^oC), and stirring speed (500 rpm) were precisely controlled, and the whole process was performed under N_2 atmosphere. The resultant co-precipitated particles were filtered, washed with distilled water for several times to remove the imprities like Na^+ and SO_4^{2-} ions and dried at 100 °C for 12 h. The obtained precursor powder was thoroughly mixed with different amounts of Li_2CO_3 (Li:(0.2Ni+0.6Mn) = 0.00, 0.08, 0.24, …, 1.52) by a dry-grind and a wet-milling ethanol process. Finally, the mixture was heated preheated at 550 °C for 6 h and subsequently calcined at 850 °C for 12 hours through a conventional furnace with air atmosphere to obtain the materials with various lithium concentrations. For comparison with $Li_{1.2}Ni_{0.2}Mn_{0.6}O_2$, the samples are correspondingly labelled as L0.00, L0.08, L0.24, …, L1.52, respectively.

Synchrotron radiation diffraction (SRD)

Ex situ SRD data of samples (i.e. L0.00-L1.52 and the electrodes) and *in situ* SRD results of coin cell (CR2025) with L0.40, L0.88 and L1.28 electrode were collected at the high-resolution Materials Science and Powder Diffraction (MSPD) beamline at ALBA, Spain, using synchrotron radiation with an energy of 30 keV. The exposure time for *ex situ* samples and *in situ* measurents was 10 and 60 s, respectively. The SRD patterns were collected using a MYTHEN 1D Position Sensitive Detector. The high-temperature SRD experiments were performed at beamline P02.1, storage ring PETRA-III at DESY (Deutsches Elektronensynchrotron) in Hamburg, Germany, using synchrotron radiation with an energy of 60 keV with an exposure time of 60 s. The prepared L0.00 was mixed with a desired amount of Li_2CO_3. The quartz capillary with the mixture was heated in a ceramic oven, in air, from room temperature to 850 °C. The diffraction patterns at P02.1 beamline were acquired using a two dimensional (2D) flat panel detector (Perkin-Elmer amorphous-Silicon detector) with a sample-to-detector distance of ∼ 1600 mm. The resulting 2D images were then integrated to one dimensional diffraction patterns by using the X-ray image processing program Fit2D. The *ex situ* powder diffraction experiments of selected eletrodes were also performed at beamline P02.1, with an exposure time of 180 s. All the cycled materials were measured in the discharged state.

Neutron powder diffraction (NPD)

The NPD measurements were performed at the high-resolution powder diffractometer SPODI, research neutron reactor MLZ/FRM II in Munich, Germany, at ambient temperature. The monochromatic neutrons were obtained at a 155° take-off angle using the 551 and 331 reflections of a vertically focusing Ge monochromator. The vertical position-sensitive multidetector consisting of 80 3He tubes with an effective height of 300 mm covering an angular two-theta range from 0° to 160° was used for data selection. All experiments were carried out in the Debye–Scherrer geometry with an incident neutron beam with a rectangular

cross section at the sample position of 40 × 30 mm^2. The powder was filled into a cylindrically thin-wall vanadium container with diameter of 10 mm. Rietveld refinements were performed on the SRD and NPD patterns to obtain the lattice parameters and phase fraction of the samples using the FullProf software package.

Pair distribution function (PDF)

PDF analysis were performed at room temperature using the instrument at beamline P02.1 at DESY, Germany. For the PDF analysis, total scattering data were collected using a short sample-to-detector distance (∼ 400 mm). The 2D X-ray scattering images were processed by using FIT2D software, and PDF data were obtained within PDFgetX2.

X-ray absorption spectroscopy (XAS)

Ex situ X-ray absorption spectroscopy (XAS) measurements were performed at XAS beamline of synchrotron radiation source at KIT, and beamline P64 at PETRA III, Germany. *In situ* XAS data of a L1.28/Li coin cell was obtained at the CLAESS beamline at ALBA synchrotron Light facility (Barcelona, Spain) [4]. The XAS data were recorded at the Mn K-edge (6539 eV), Ni K-edge (8333 eV) in transmission mode, respectively. X-ray absorption near edge spectra (XANES) region of the XAS spectrum were acquired by subtracting the pre-edge background from the overall absorption and normalizing to the spline fit using the ATHENA software package.

Magic angle spinning nuclear magnetic resonance (MAS-NMR) spectroscopy

Solid-state ^7Li MAS-NMR experiments were performed on a Bruker Avance 200 MHz spectrometer equipped with a 1.3 mm MAS probe at a spinning speed of around 60 kHz. The magnetic field strength was 4.7 T together with a rotor-synchronized Hahn-echo pulse sequence.

Scanning electron microscopy (SEM)

The morphology of the materials was detected by a Zeiss Merlin scanning electron microscope (SEM) using an acceleration voltage of 10 keV.

Chemical analysis

The concentration of chemical compounds Li, Ni, Mn was determined by Inductively coupled plasma optical emission spectroscopy (ICP-OES, OPTIMA 4300 DV, PerkinElmer). The samples were dissolved in a mixture of hydrochloric and hydrofluoric acid at 235 °C for 3 h with the pressure digestion system DAB 2 (Berghof). Oxygen was determined by carrier gas hot extraction, which is known as the insert gas fusion method (LECO TC 600).

Bragg X-ray coherent diffraction imaging (BCDI)

BCDI measurements were performed at the ID01 beamline, ESRF – The European Synchrotron [5]. An 8 keV coherent focused X-ray beam (500 × 500 nm FWHM) was used to illuminate a single crystallite from the L1.28 coin cell. The rocking curve was obtained by scanning the x-ray energy (± 500 eV) [6] in 10eV steps, with an exposure time of 2.5 s per point, the focusing optic (KB mirror) being achromatic to ensure the sample remained in the beam at all times. A maxipix detector was placed 0.896 m from the sample, at the anticipated layered 001_m bragg peak ($2\theta = 18.756°$). The diffraction pattern was oversampled by a factor 3 in all dimensions after binning, see Supplementary Figure 10. The phase retrieval procedure, executed with the PyNX python package (PyNX python library, https://gitlab.esrf.fr/favre/PyNX (2019)) [7], included 100 iterations of the RAAR algorithm followed by a support optimisation with shrinkwrap, repeated 10 times, a further 300 iterations of HIO, and 50 iterations of ER to finish. A total of 10000 independent phasing cycles were made, the best 100 were chosen based on the lowest free log-likelihood and

combined by computing their eigenvectors. In this case the best eigen solution has a weight of 78.2% and the phase retrieval transfer function computed [8] as shown in Supplementary Figure 10 indicates an average spatial resolution of 35 nm in 3D. The dataset was postprocessed using a BCDI python package (https://github.com/carnisj/bcdi (2019) 10.5281/zenodo.3257617). Examples of the reconstructed object at each stage of the postprocessing is shown in Supplementary Figure 12, best eigen solution, phase ramp removal and offset, refraction and absorption correction ($\delta = 1.5558 \times 10^{-6}$, $\beta = -1.5810 \times 10^{-7}$). The distribution of lattice parameters relative to the reference 001_m is shown in Supplementary Figure 13.

Electrochemical Characterization

The electrochemical performances of the prepared electrode materials were carried out by galvanostatic cycling in a coin-type half-cell (CR2032). The coin cells were assembled in an argon filled glove box (MBraun) with lithium metal (15.6 mm diameter, 250 μm thickness) as anode, 200 μL LP30 electrolyte and two layers of Celgard 2325 membrane as separator. A mixture of the obtained Mn-rich oxide powder (80 wt. %), carbon black (13 wt. %, TIMCAL, Super C65) and polyvinylidene fluoride (PVdF, 7 wt. %, Solef 6020, Solvay) was mixed with N-Methyl-2-pyrrolidone (NMP, Sigma-Aldrich), the obtained slurry was coated on aluminum foil. This film was fried at 80 °C for 12 hours and discs were punched out with a diameter of 12 mm to obtain the positive elelctrode. The mass loading of the active material in the cathode was 3–4 mg cm^{-2}. The electrolyte used for the cells cycled in the voltage range of 2.0–4.8 V was LP30 (1 M LiPF6 in EC:DMC (50:50, wt.), BASF). The half-cell measurements were conducted at a current density of C/10 (1 C = 320 mA g^{-1}) at 25 °C using a VMP3 multi-channel potentiostat (Bio-Logic, France).

Supplementary References

1. Tarascon, J. M., Wang, E., Shokoohi, F. K., McKinnon, W. R. & Colson, S. The spinel phase of $LiMn_2O_4$ as a cathode in secondary lithium cells. *J. Electrochem. Soc.* **138,** 2859–2864 (1991).
2. Seo, D., Lee, J., Urban, A., Malik, R., Kang, S. & Ceder, G. The structural and chemical origin of the oxygen redox activity in layered and cation-disordered Li-excess cathode materials. *Nat. Chem.* **8,** 692–697 (2016).
3. Sathiya, M. *et al.* Origin of voltage decay in high-capacity layered oxide electrodes. *Nat. Mater.* **14,** 230–8 (2015).
4. Simonelli, L. *et al.* CLÆSS: The hard X-ray absorption beamline of the ALBA CELLS synchrotron. *Cogent Phys.* **3,** 1231987 (2016).
5. Leake, S. J. *et al.* The nanodiffraction beamline ID01/ESRF: A microscope for imaging strain and structure. *J. Synchrotron Radiat.* **26,** 571–584 (2019).
6. Cha, W. *et al.* Three Dimensional Variable-Wavelength X-Ray Bragg Coherent Diffraction Imaging. *Phys. Rev. Lett.* **117,** 1–5 (2016).
7. Mandula, O., Elzo Aizarna, M., Eymery, J., Burghammer, M. & Favre-Nicolin, V. PyNX.Ptycho: A computing library for X-ray coherent diffraction imaging of nanostructures. *J. Appl. Crystallogr.* **19,** 1842–1848 (2016).
8. Favre-Nicolin, V., Leake, S. % Chushkin, Y. Free log-likelihood as an unbiased metric for coherent diffraction imaging. *arXiv Prepr. arXiv1904.07056* (2019).
