# Supplementary material for: Structural insights into the formation and voltage degradation of lithium- and manganese-rich layered oxides
Source: Nat Commun. 2019 Nov 26;10:5365. doi: 10.1038/s41467-019-13240-z (PMC6879514; doi:10.1038/s41467-019-13240-z)
Supplement: Supplementary file 3 — Description of Additional Supplementary Files [file 41467_2019_13240_MOESM3_ESM.pdf]

Description of Additional Supplementary Files

Supplementary Movie 1 Animation: Strain at the surface of a single particle of the L1.28 electrode after cycling 868 times over one year. The BCDI reconstructed strain corresponds to the component along the Y axis which is parallel to the momentum transfer vector Q.

Supplementary Movie 2 Animation: Three orthogonal cross sectional views of the strain in a single particle of the L1.28 electrode determined by BCDI after cycling 868 times over one year. The given strain component is the projection onto the normal of the respective cross sectional plane.
